# Detection of extended spectrum beta lactamases (ESBLS) and carbapenemases in Escherichia coli isolated from cow-dungs and poultry droppings

**DOI:** 10.1186/2047-2994-4-S1-P142

**Published:** 2015-06-16

**Authors:** ES Olivia, I Yusuf, SA Ahmad

**Affiliations:** 1 Microbiology, Delta State University, Abraka, Nigeria; 2 Microbiology, Bayero University, Kano, Kano, Nigeria; 3 Microbiology, Universiti Putra Malaysia, Selangor, Malaysia; 4 Biochemistry, Universiti Putra Malaysia, Selangor, Malaysia

## Introduction

The rising needs for source of protein has led to uncontrol use of antibiotics in cattles and poultry farms in Nigeria. There is great concern among researchers in the region on possibility of acquiring multi drug resistant bacteria from their meats directly or from plants when their wastes are used as manure. Equally possible is spread of the multi drug resistant bacteria by farm workers which are know for poor sanitation.

## Objectives

The study was carried out to determine if extended spectrum beta-lactamse (ESBLs) and carbapenemase can be detected in *E. coli* isolated from cow-dungs and poultry droppings from different farms located in Abraka-Delta, South-South geo-political zone of Nigeria.

## Methods

A total of 146 and 88 *E.coli* were isolated from cow-dungs and poultry droppings respectively. Antimicrobial susceptibility test was carried out by the disk diffusion methods using ceftazidime (30µg), cefuroxime (30µg), cefotaxime (30µg), cefixime (5µg), gentamycin (100µg), amoxicillin-clavulanic acid (30µg), ciprofloxacin (5µg), ofloxacin (5µg), trimethoprime- sulfamethoxazole (25µg), nitrofurantion (300µg), and meropenen (10µg). ESBL and carbapenemase production were determined by double disc synergy test (DDST) and Modified Hodge’s test respectively. Carbapenemase producers were further screened for metallo-beta lactamases (MBL) production by double disc synergy test.

## Results

Isolates from cow-dungs were highly resistant to amoxicillin-clavulanic acid (94.5%), cefixime (89.0%), cefotaxime (82.2%), cefuroxime (80.8%), and ceftazidime (78.1%). All isolates from poultry droppings were resistant to amoxicillin-clavulanic acid and cefotaxime (100%). Higher prevalence for ESBLs (27.27%), carbapenemase (18.18%) and MBLs (17.04%) production was found in the isolates obtained from poultry droppings than isolates from cow dungs which recorded 23.97%, 8.21% and 6.85% for ESBLs, carbapenemase and MBL production respectively.

## Conclusion

The detection of ESBL producing and carbapenem hydrolysing isolates in cow-dungs and poultry droppings obtained from the environment is highly worrisome because these dungs/droppings are used as manures in ponds and vegetables. Evidence abounds on their transmission to humans.

## Disclosure of interest

None declared.

